# Engineering of a Carbonic Anhydrase from *Hydrogenimonas thermophila* Through Fusion Tags and Surface Mutagenesis Enhances Solubility While Revealing Stability–Function Relationships

**DOI:** 10.3390/ijms27167498

**Published:** 2026-08-21

**Authors:** Colleen Varaidzo Manyumwa, Carsten Jers, Ivan Mijakovic

**Affiliations:** 1 Novo Nordisk Foundation Biotechnology Research Institute for the Green Transition, Technical University of Denmark, 2800 Kongens Lyngby, Denmark; covama@dtu.dk (C.V.M.); cjer@dtu.dk (C.J.); 2 Department of Life Sciences, Chalmers University of Technology, SE-41296 Gothenburg, Sweden

**Keywords:** alpha carbonic anhydrase, protein solubility, solubility tag, *Hydrogenimonas thermophila*, thermostability, rational engineering, hydrothermal vent, CO_2_ hydration, protein engineering, mutagenesis

## Abstract

Protein solubility can limit enzyme performance in industrial applications. This is the case for some carbonic anhydrases (CAs), key enzymes for CO_2_ capture and utilization. In this study, we investigated an α-class CA from the thermophilic bacterium *Hydrogenimonas thermophila* (HtCA), which was predominantly expressed as an insoluble protein in Escherichia coli. Surface analysis using Molecular Operating Environment (MOE) revealed extensive hydrophobic regions, suggesting a basis for its poor solubility. To improve solubility, three C-terminal fusion tags were evaluated (Gb1, ng3-NEXT, and T7B9). All tagged variants showed markedly increased soluble expression as determined by sodium dodecyl sulfate–polyacrylamide gel electrophoresis (SDS-PAGE) analysis. To reduce surface hydrophobicity, selected residues were substituted with charged amino acids. Most variants displayed improved solubility, and V136D showed enhanced thermostability, retaining 76% activity after exposure to 90 °C for an hour. However, the F177D variant completely lost all enzymatic activity, highlighting the importance of evaluating both solubility and catalytic function during protein engineering. Molecular dynamics simulations supported the experimental findings, revealing that thermostable variants exhibited reduced structural fluctuations and favorable free-energy landscapes, while the inactive F177D mutant sampled a broader conformational space and higher-energy conformations, consistent with decreased structural stability and loss of catalytic activity.

## 1. Introduction

Protein solubility is a fundamental determinant of protein functionality and applicability in both biological research and industrial biotechnology [1]. At its core, solubility reflects the equilibrium between soluble and insoluble protein states and is governed by intrinsic factors such as amino acid composition, surface charge distribution, and hydrophobicity, as well as extrinsic conditions including pH, ionic strength, temperature, and the presence of cofactors [2]. Perturbations in these conditions frequently lead to protein aggregation or precipitation, often resulting in loss of activity and reduced experimental reproducibility.

A range of strategies have been developed to enhance protein solubility. Fusion to solubility-enhancing tags is widely used to improve folding efficiency and prevent aggregation during expression. Tags that are commonly used include maltose-binding protein (MBP), glutathione S-transferase (GST) and thioredoxin (Trx) [3,4,5,6]. More recently reported solubility-enhancing tags include the NT11, XXA and Spy tags [7,8,9,10]. In addition, rational protein engineering approaches, including site-directed mutagenesis of surface residues, aim to increase net charge, reduce hydrophobic surface patches, or introduce favorable electrostatic interactions [11,12]. Several computational tools, including Aggrescan3D, AggrescanAI, CamSol, and Protein-Sol, have been developed to predict aggregation-prone regions and guide the rational design of more soluble protein variants [13,14,15,16,17]. Emerging AI-based tools, including PROTSOLM, PLMSol, and SOuL_MuSiC, can predict protein solubility and assess protein solubility changes in computationally generated variants [18,19,20]. Other approaches include co-expression with molecular chaperones, optimization of expression conditions, and buffer engineering using additives such as glycerol to stabilize proteins in solution [21,22,23]. Together, these methods provide a versatile toolkit for addressing solubility limitations, though improvements must often be balanced against potential impacts on protein activity and stability.

Carbonic anhydrases (CAs) represent a particularly relevant system in this context. These metalloenzymes catalyze the reversible hydration of carbon dioxide to bicarbonate and protons, a reaction that is fundamental to physiological processes such as respiration, pH regulation, and ion transport, as well as industrial applications including carbon capture and sequestration [24,25,26]. α-CAs, in particular, have attracted significant attention for both fundamental studies and biotechnological applications due to their high activity and well-characterized structures [27,28]. However, like many enzymes, certain α-CA variants can exhibit limited solubility under recombinant expression conditions or in non-native environments, constraining their practical use [29]. This is especially relevant in applications such as carbon capture, where enzymes must function efficiently under high CO_2_ concentrations, varying pH, and elevated temperatures.

In this study, we investigated strategies to enhance the solubility of an α-CA from a hydrothermal vent bacterium, *Hydrogenimonas thermophila* (HtCA), which exhibited low purification yield and clear signs of aggregation. This observation was consistent with its highly hydrophobic surface profile viewed using Molecular Operating Environment (MOE) [30]. Overexpression of recombinant proteins in *Escherichia coli* has commonly resulted in the formation of insoluble inclusion bodies, necessitating complex refolding procedures that are often inefficient and yield low amounts of functional protein [31]. We therefore made use of three proven solubility tags, ng3-NEXT tag [29,32,33], T7B9 tag [34,35,36] and Gb1 tag [37,38,39,40], and through this study, we revealed that the addition of solubility tags would improve the solubility of HtCA, thus improving its purification yield. We also uncovered the benefits of mutating surface residues to decrease surface hydrophobicity and thereby improve protein solubility. However, some surface mutations had unintended effects on protein stability and catalytic function. Molecular dynamics (MD) simulations were further used to investigate how these mutations influence protein dynamics and stability. Together, these experimental and computational analyses provide insights into the rational engineering of more soluble and robust CAs.

## 2. Results

### 2.1. Surface Hydrophobicity Reveals Potential Drivers of Poor Soluble Recovery of HtCA

**Figure 1:**
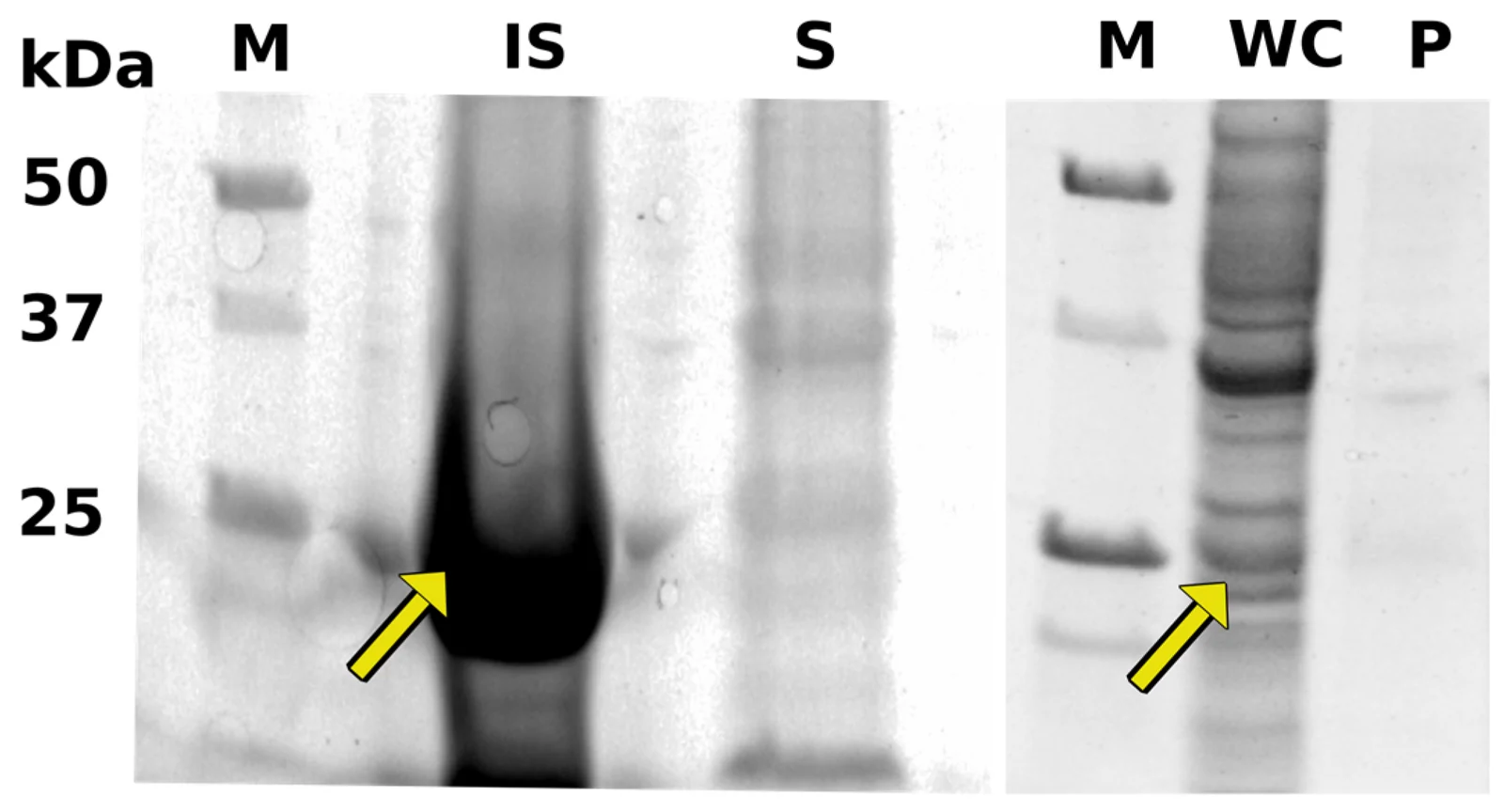
SDS-PAGE showing HtCA in IS—insoluble fraction, S—soluble fraction, WC—whole cell fraction, and P—purified fraction. The yellow arrows indicate the protein bands corresponding to HtCA’s predicted molecular weight.

A codon-optimized gene encoding HtCA was cloned into the pETM10 expression vector and transformed into *Escherichia coli.* Initial expression trials revealed by SDS-PAGE that the majority of the recombinant protein was present in the insoluble fraction following cell lysis and centrifugation, suggesting protein aggregation and limited soluble expression (Figure 1). Consistent with this observation, limited protein was recovered following purification. Consequently, computational analyses were undertaken to investigate potential structural factors contributing to this behavior.

**Figure 2:**
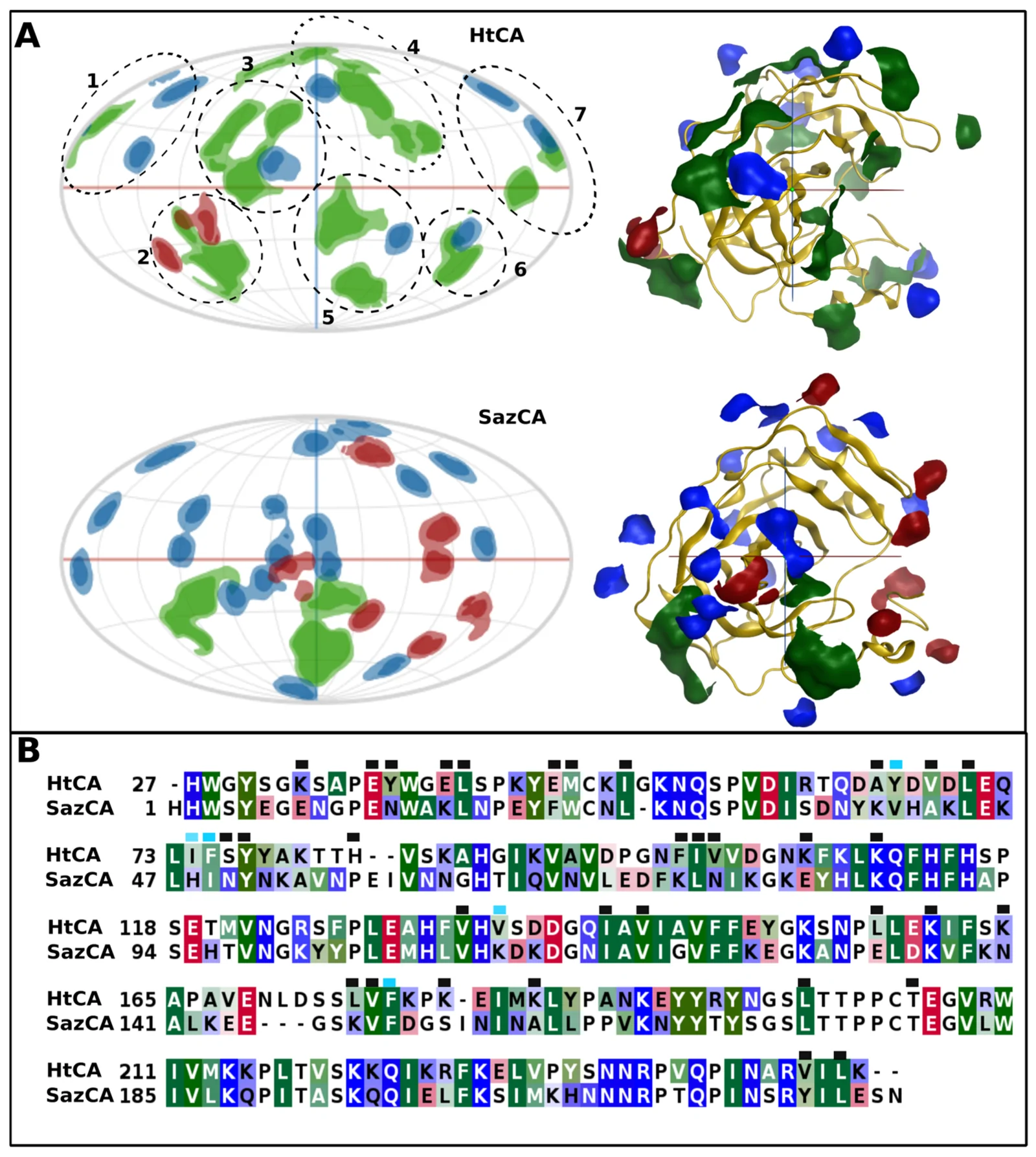
(**A**) Surface patch representation of the protein structures for CAs from *H. thermophila*, HtCA (top) and *S. azorense*, SazCA (bottom). Patches are colored as follows: green—hydrophobic patch; red—negatively charged patch; blue—positively charged patch. Dotted circles denote the surface patch clusters, numbered 1 to 7, whose residues are listed in Table 1. (**B**) Sequence alignment between CAs from *H. thermophila* and *S. azorense*, colored by percentage identity, with darker shading corresponding to higher identity. Residues are colored as in A: green—hydrophobic residues, red—negatively charged residues; blue—positively charged residues. Residues with rectangles above indicate the residues in the hydrophobic surface patches identified by MOE. The cyan-colored ones show the residues chosen for mutagenesis.

Surface characterization of HtCA and the highly efficient CA from *Sulfurihydrogenibium azorense,* SazCA [41] was performed using MOE to evaluate the distribution of hydrophobic and charged regions. MOE is a molecular modeling software package which can be used to identify and quantify hydrophobic and electrostatic surface patches on proteins. The 2D surface projection of HtCA displayed extensive and continuous hydrophobic surface patches (Figure 2A). These hydrophobic regions were more prominent and less interrupted by charged residues compared to SazCA. The surface-exposed residues associated with these clusters are listed in Table 1. Additionally, HtCA showed a less uniform distribution of charged residues, with fewer positively charged regions observed in proximity to hydrophobic clusters. This resulted in a surface with predominantly hydrophobic character, particularly in specific localized regions. In contrast, SazCA exhibited a relatively balanced surface composition, with hydrophobic regions interspersed among positively and negatively charged patches (Figure 2A). These features were distributed across the protein surface without forming large continuous clusters.

Given that the surface analysis was carried out on monomeric structures, while α-CAs are known to dimerize in solution, interface residues for HtCA were identified and annotated in Table 1, and they were excluded from subsequent mutational investigations to avoid perturbation of dimer formation [42].

Furthermore, Aggrescan3D [15], which predicts aggregation-prone surface regions based on residue aggregation propensities and structural context, identified I74, F75 and F177 as the residues with the highest aggregation scores (Supplementary Figure S1). Their location within the two largest exposed hydrophobic patch clusters (3 and 4) further supported their selection for mutagenesis. Y66 and V136 were also chosen to disrupt patch clusters 2 and 5, respectively. Given the relative absence of negatively charged surface patches on HtCA, selected residues were mutated to aspartate (D) or glutamate (E) to increase surface charge and potentially reduce aggregation.

### 2.2. Surface Mutations and Fusion Tags Improve Soluble Expression of HtCA

**Figure 3:**
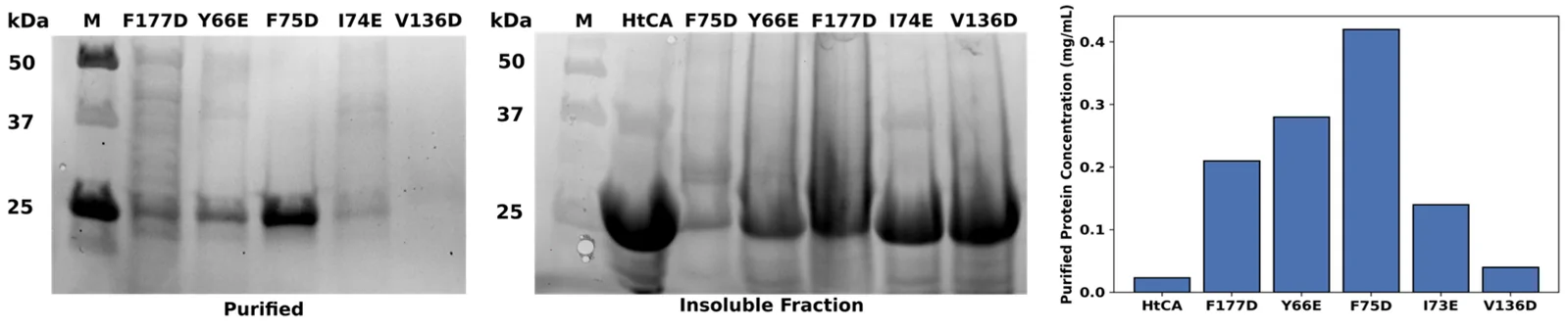
SDS-PAGE showing proteins HtCA variants (25 kDa) in the purified fractions (Gel 1) and insoluble cell fractions (Gel 2). Bar graph shows concentrations of the purified fractions in mg/mL.

Mutations were introduced at the residue positions highlighted in the sequence alignment (Figure 2B) and indicated in Table 1 using the vector encoding non-tagged HtCA. Site-directed mutagenesis targeting hydrophobic surface residues yielded variable results. Among the variants tested, mutations such as Y66E, I74E, F75D and F177D (Figure 3) resulted in increased soluble protein levels compared to HtCA. This is evidenced by enhanced band intensity in the purified samples on SDS-PAGE gels. Variant F75D in particular had 18 times more soluble protein in the purified fraction, with a protein concentration of 0.42 mg/mL. In contrast, V136D did not show much improvement in solubility and remained predominantly in the insoluble fraction.

**Figure 4:**
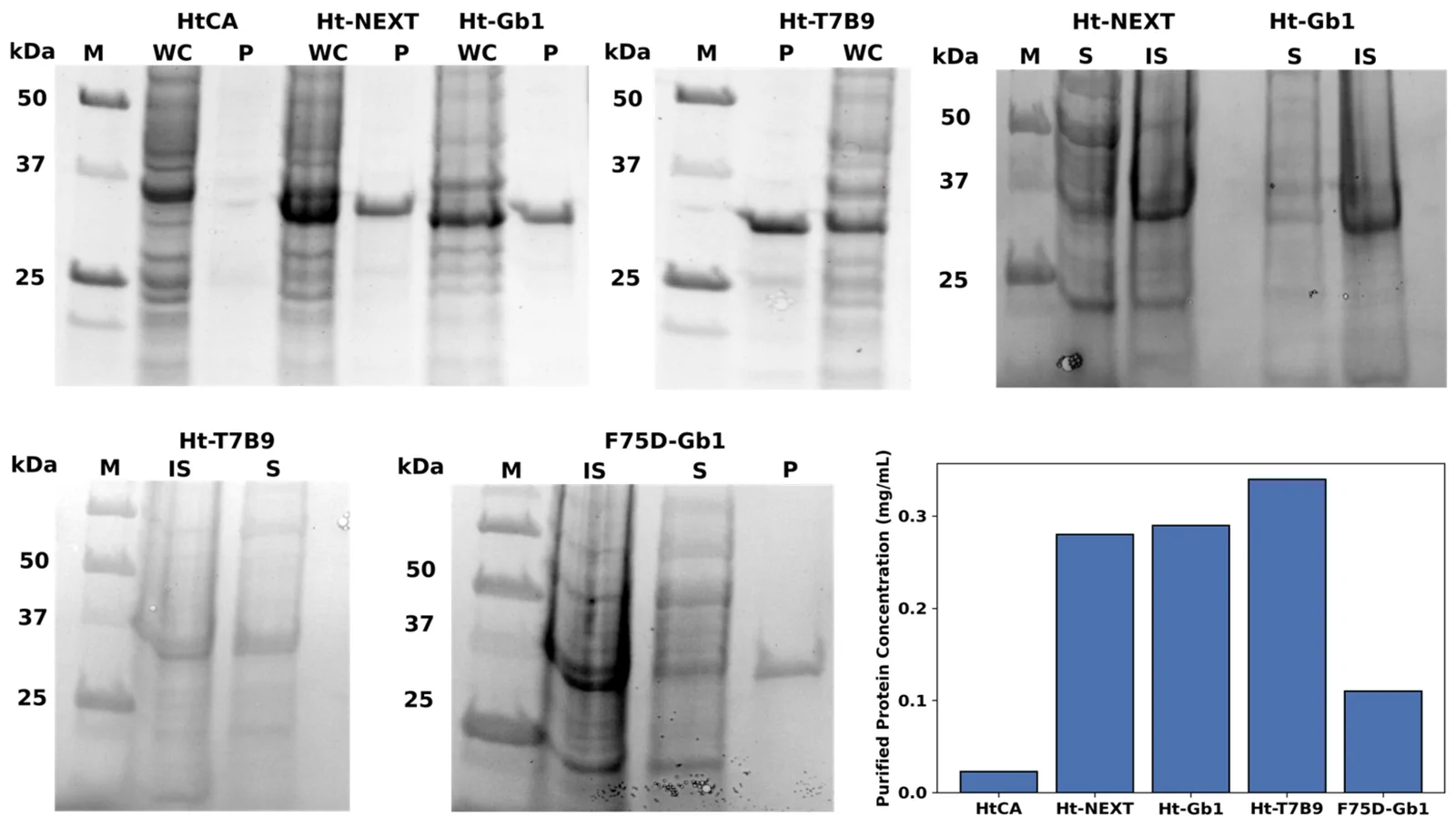
SDS-PAGE showing tagged-HtCA variants; Ht-NEXT (33 kDa), Ht-Gb1 (32.8 kDa), Ht-T7B9 (31.1 kDa) and F75D-T7B9. Fractions shown are WC—whole cell fraction, P—purified fraction, S—soluble fraction, and IS—insoluble cell fraction. Bar graph shows concentrations of the purified fractions in mg/mL.

In the vector, we subsequently inserted DNA fragments encoding the solubility tags T7B9, ng3-NEXT and Gb1. Upon induction of HtCA synthesis in the various strains, we evaluated the performance by SDS-PAGE (Figure 4). All fusion-tagged constructs displayed increased protein levels in the soluble fraction, decreased protein levels in the insoluble fraction, and were readily detectable after purification. The amount of insoluble HtCA also decreased significantly compared to HtCA. Variant F75D was then coupled with the T7B9 tag to assess whether the solubility-enhancing effects of surface engineering could be further augmented by fusion tagging. This, however, interestingly yielded lower improvements in soluble expression than just the tag or the mutation alone.

### 2.3. Mutations Reduce Enzymatic Activity While Increasing Thermostability

**Figure 5:**
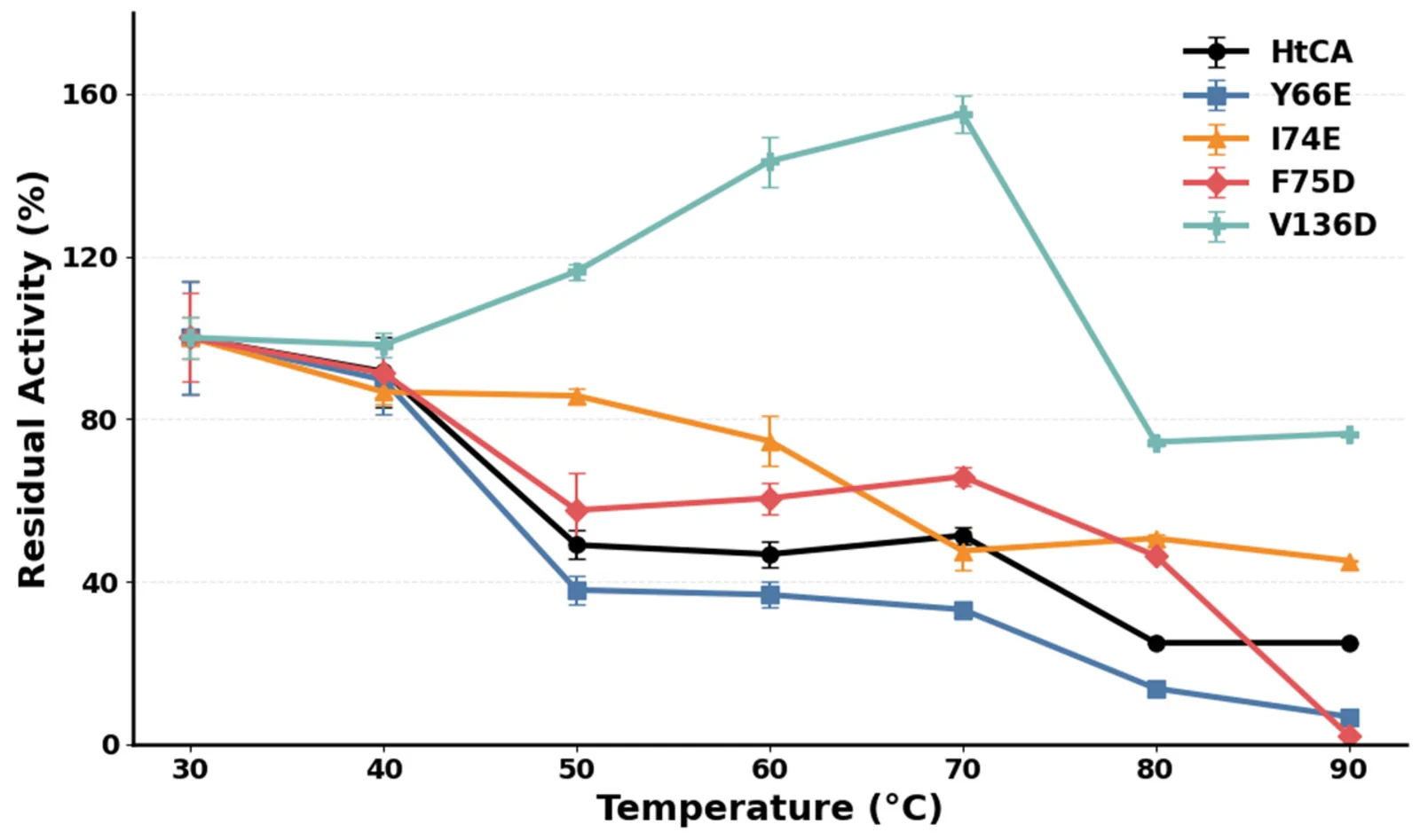
Thermostability profiles of HtCA and variants following thermal incubation. Residual activity was expressed as a percentage relative to the non-heated control (100% activity). Data are presented as mean ± SD from three independent replicates (*n* = 3). HtCA exhibited a progressive reduction in residual activity with increasing temperature, retaining approximately 25% activity at 90 °C. Among the variants tested, Y66E showed lower thermostability than HtCA across most temperatures, with a substantial decline in activity observed above 70 °C. In contrast, the variants I74E and V136D demonstrated improved thermal tolerance relative to HtCA, with I74E retaining approximately 45% residual activity at 90 °C. Notably, V136D retained higher residual activity across the entire temperature range and exhibited increased activity between 50 °C and 70 °C, reaching a maximum residual activity of approximately 155% at 70 °C.

The catalytic activity of HtCA variants was evaluated using the CO_2_ hydration assay. HtCA exhibited measurable activity under the assay conditions (560 WAU/mg). I74E, F75D, and Y66E resulted in a significant decrease of almost 80% in specific enzymatic activity relative to HtCA. F177D completely lost activity, while V136D retained over 50% of HtCA specific activity. To further assess the impact of mutations on protein stability, thermostability of HtCA and selected variants was evaluated by measuring residual enzymatic activity following incubation at temperatures ranging from 30 °C to 90 °C for 1 h (Figure 5). Since variant F177D exhibited no detectable enzymatic activity, it was excluded from further thermostability analysis and graphical representation.

### 2.4. Mutations Alter Structural Stability and Residue-Level Flexibility

To investigate structural dynamics, MD simulations were performed for HtCA and its variants over 100 ns at 300 K. Root-mean-square deviation (RMSD), radius of gyration (Rg), and root mean square fluctuations (RMSFs) were used to evaluate the conformational stability, structural compactness, and dynamic flexibility of HtCA and its variants, providing insights into the effects of mutagenesis on thermostability and activity.

**Figure 6:**
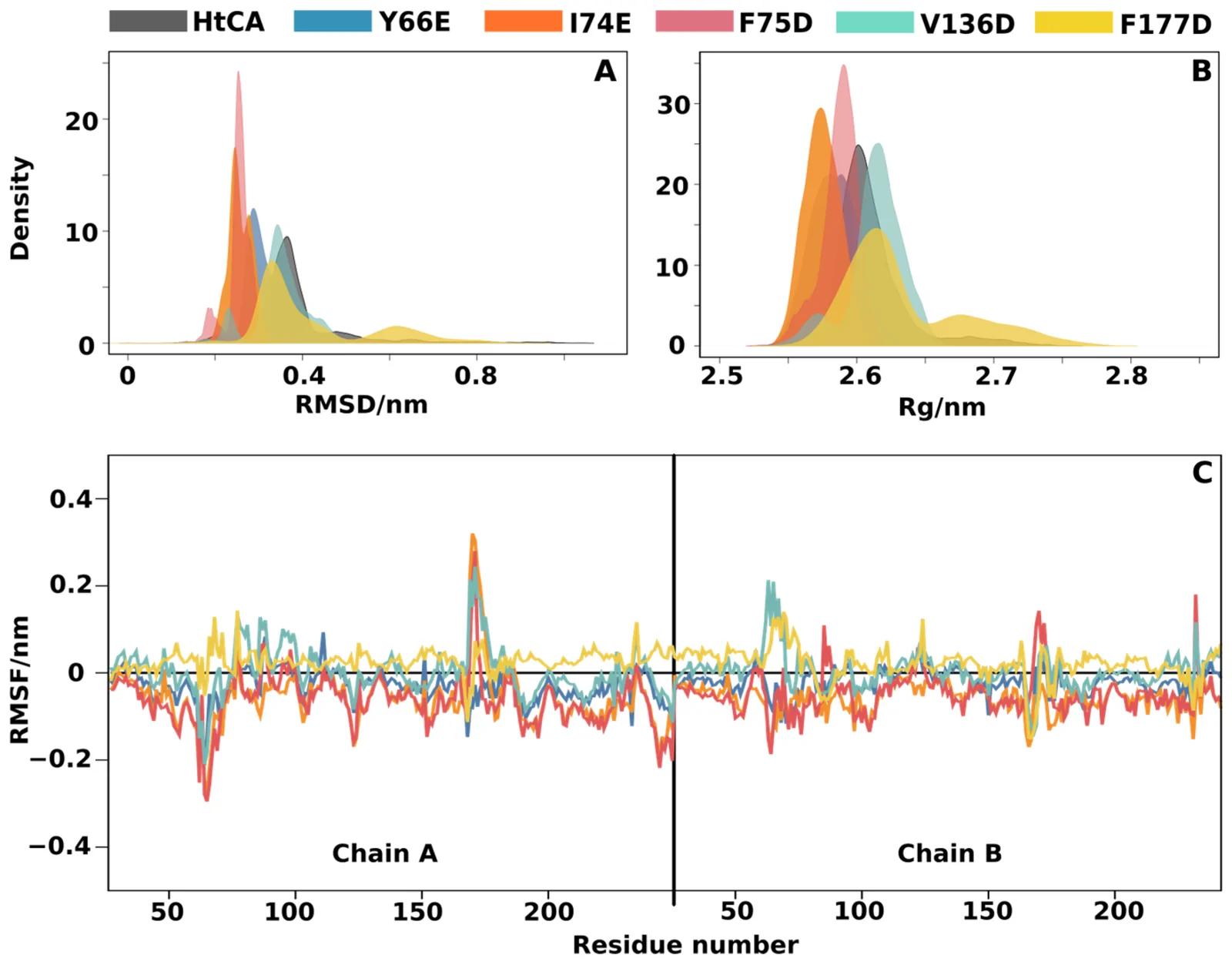
Kernel density plots (KDE) showing (**A**) backbone RMSD and (**B**) backbone Rg distributions of HtCA and variants during 100 ns MD simulations. RMSD distributions reflect overall structural stability and conformational deviations while Rg distributions indicate changes in protein compactness. (**C**): Per-residue RMSF differences between HtCA and variants, highlighting mutation-induced changes in local residue flexibility. The HtCA profile is shown as the zero reference line; positive and negative values indicate increased and decreased flexibility relative to HtCA, respectively.

RMSD distributions for Y66E and V136D remained within a similar range to HtCA (Figure 6). Variants I74E and F75D exhibited less conformational variability shown by a narrower density plot. F177D displayed broader distributions and transient increases in deviation, suggesting greater conformational variability and structural rearrangements during the simulation period.

Analysis of the Rg revealed relatively small differences in global compactness between HtCA and variant proteins, with slightly more compactness observed in I74E and F75D. However, similar to RMSD, F177D exhibited broader Rg distributions and slightly elevated values, correlating to increased conformational sampling and indicating reduced structural compactness relative to HtCA.

RMSF profiles highlight a decrease in overall fluctuation patterns for most of the variants compared to HtCA. I74E and F75D showed the most decrease in residue fluctuations. F177D showed an increase in RMSF for most of its residues. Several regions exhibit distinctly different mobility in the variants relative to HtCA, indicating localized changes in dynamics rather than global destabilization. RMSF increased for residues 169–176, which is a loop region, for all variants except Y66E, where RMSF decreased. Loop with residues 61–69 had the most decrease in fluctuations for all mutants except F177D.

### 2.5. Surface Mutations Alter the Conformational Energy Landscapes of HtCA Variants

**Figure 7:**
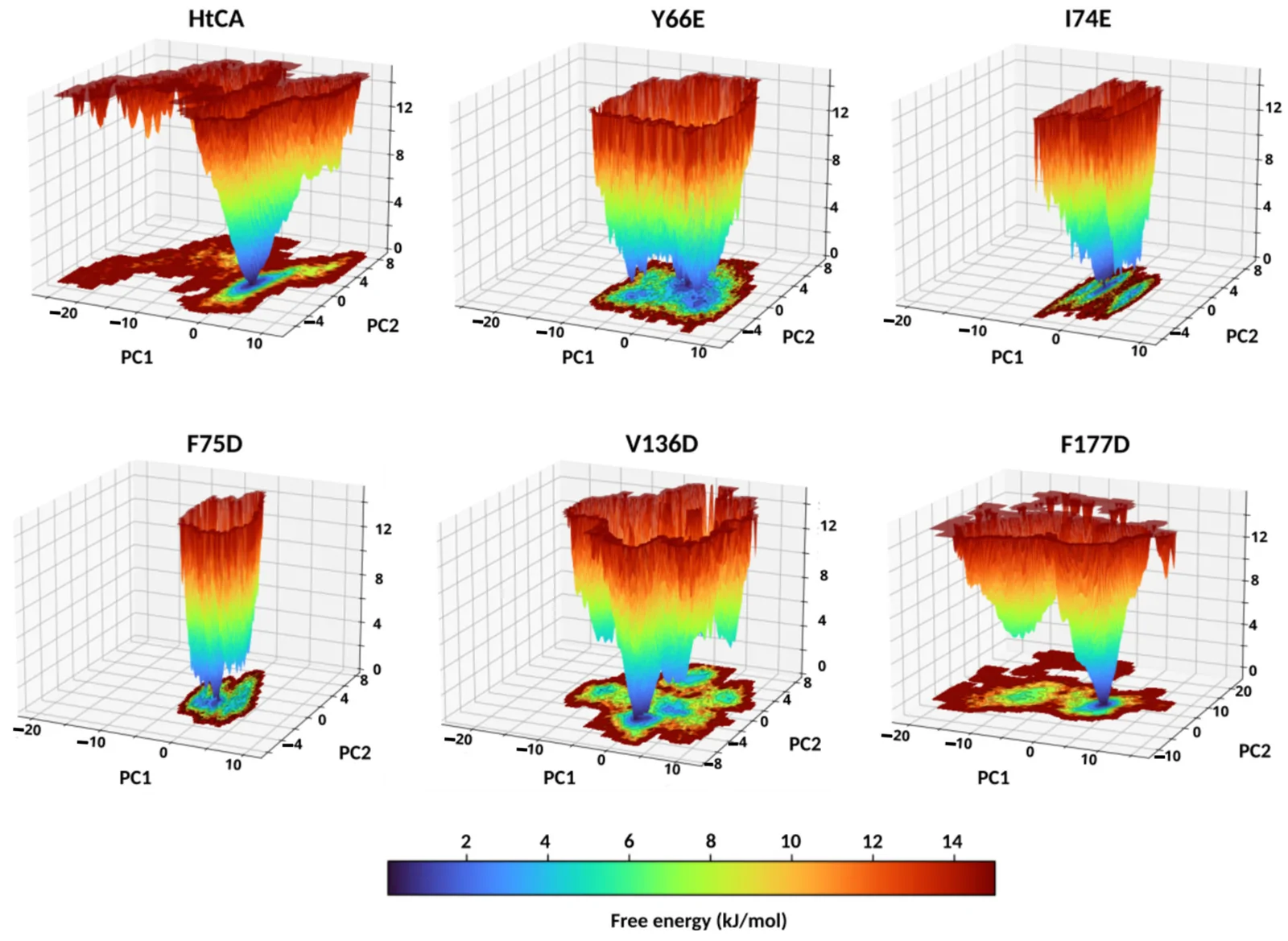
Free energy landscape (FEL) of the protein conformational ensemble projected onto the first two principal components (PC1 and PC2). The color scale represents relative free energy (kJ/mol), where darker/lower-energy regions correspond to more populated and thermodynamically favorable conformational states, while higher-energy regions indicate less populated conformations. Minima on the landscape represent stable conformational basins sampled during the MD simulation. Differences in the number, distribution, and depth of these basins reflect mutation-induced changes in the conformational landscape and the relative stability of the sampled protein conformations.

Free energy landscapes (FELs) were constructed along the first two principal components (PC1 and PC2) which represent the dominant collective motions captured from the trajectory. This was done to characterize the conformational states sampled by HtCA during the simulation, providing insights into the energetic stability of different conformations and how these states were altered by mutagenesis, with potential implications for thermostability and activity (Figure 7).

HtCA exhibits a single, well-defined low-energy basin, indicative of a dominant conformational state, but with higher energy states observed as well. In contrast, F177D displays a pronounced, funnel-like basin comparable in depth to HtCA, indicating the presence of a dominant low-energy conformation despite mutation. The other variants showed more heterogeneous landscapes. Y66E and I74E show reduced but more diffuse basins, suggesting increased conformational variability. F75D retains a coherent low-energy basin in a highly reduced landscape, indicating low conformational sampling. V136D similarly exhibits a defined minimum with additional surrounding low-energy regions, suggesting the presence of multiple accessible substates. Overall, the FELs reveal mutation-dependent differences in the breadth, shape, and ruggedness of the sampled conformational space.

## 3. Discussion

Hydrophobic patches on protein surfaces are known to promote aggregation by facilitating inter-molecular interactions, which can impair protein stability and function. Surface analysis of HtCA using MOE was performed on monomeric structures, as the objective was to evaluate intrinsic surface properties of the folded protein independent of quaternary assembly. The large, contiguous hydrophobic regions observed across its surface are likely to increase the tendency of HtCA to self-associate in solution. In contrast, the surface of SazCA, a very soluble CA, exhibited a more balanced distribution of hydrophobic and charged residues, potentially contributing to improved solubility and reduced aggregation. Residues within prominent hydrophobic clusters were therefore targeted for substitution with polar or positively charged amino acids to disrupt patch continuity and reduce aggregation propensity. Here, Aggrescan3D results acted as a guide for choosing some residues with the highest aggregation scores.

The incorporation of solubility-enhancing fusion tags significantly improved the soluble expression of HtCA. The Gb1 tag, derived from the B1 domain of protein G, has been widely used to enhance protein solubility through improved folding and stabilization [37]. In this study, Gb1 improved the solubility of HtCA when positioned at the C-terminus. A recent study demonstrated that fusion with the Gb1 solubility tag also substantially enhanced the soluble expression of hyperactive Tn5 transposase in *E. coli*, enabling high-yield production of functionally active protein [43]. The T7B9 tag is a 40-residue peptide derived from bacteriophage T7 gene 10B that has been reported to improve the solubility and folding of recombinant proteins during expression in *E. coli* [34,44]. Its solubilizing effect is attributed primarily to its high net negative charge (−12), which reduces protein aggregation through electrostatic repulsion [34]. This rationale was particularly relevant for HtCA, which exhibited a deficiency of negatively charged surface patches. Consistent with this hypothesis, fusion of the T7B9 tag resulted in a marked increase in the soluble expression of HtCA, supporting the role of surface charge engineering using tags in improving protein solubility. The ng3-NEXT tag, an intrinsically disordered peptide previously shown to enhance the solubility of *Thermovibrio ammonificans* CA (TaCA), also improved the solubility of HtCA [29,32]. Truncated variants of the NEXT tag were previously studied, demonstrating that the C-terminal 16 residue region, (NEXT_C16_) retained strong in vitro solubility-enhancing activity [29]. While prior studies primarily examined N-terminal fusion, our results demonstrate that C-terminal placement of the ng3-NEXT tag can also enhance solubility.

The improved soluble expression observed for the Y66E, I74E, F75D, and F177D variants suggests that introducing charged residues at selected hydrophobic surface positions can reduce aggregation and enhance protein solubility. In this instance, the limited presence of negatively charged surface patches on HtCA was addressed by introducing negatively charged residues, thereby increasing surface polarity and potentially improving interactions with the surrounding aqueous environment. The lack of improvement observed for V136D is consistent with the low aggregation propensity predicted by Aggrescan3D. These results highlight that while reducing surface hydrophobicity can be an effective strategy for improving protein solubility, not all hydrophobic residues contribute equally to aggregation propensity.

V136D’s ability to maintain activity after incubation at 90 °C is comparable to some thermostable CAs that have been previously reported. This includes PmCA from *Persephonella marina* (75% activity after 120 min at 90 °C), as well as an engineered variant CA from *Persephonella hydrogeniphila* that retained over 150% activity after 1 h at 80 °C [45,46,47]. An ultrastable CA has also been engineered from *Desulfovibrio vulgaris,* staying active after a 14-week incubation at 40 °C [48]. Although most variants exhibited improved thermostability compared to HtCA, this enhancement was not accompanied by retention of catalytic activity. In contrast, the D168K surface mutation in *Nitratiruptor tergarcus* CA was reported to improve both catalytic activity and thermostability, demonstrating that the effects of surface engineering on stability and function can be highly mutation- and protein-dependent [49]. To gain further insight into the structural basis of the stability–function relationship, MD simulations for HtCA and the variants were analyzed using RMSD, Rg, RMSF and FEL analysis.

Compared to HtCA, the combined RMSD and Rg analyses suggest that none of the mutations, except for F177D, induce large-scale unfolding or disrupt the overall protein fold. The RMSF results for I74E and F75D were consistent with the RMSD and Rg analyses, with narrower density peaks compared to HtCA, indicating reduced conformational sampling and lower residue fluctuations. In particular, loops 62–69, which showed the greatest RMSF decrease in variants I74E, F75D, and V136D, is located at the protein interface, suggesting that these variants possess a more stable interfacial region. This may have contributed to the increased thermostability observed experimentally. I74E and F75D retained well-defined low-energy basins while sampling a relatively restricted conformational space. Furthermore, I74E sampled two distinct low-energy conformations, indicating the presence of multiple stable substates within the folded ensemble. The most thermostable variant, V136D, exhibited several low-energy basins, which may suggest that enhanced thermostability does not necessarily arise from increased rigidity, but may instead reflect stabilization of multiple closely related conformational substates.

In contrast, the inactive F177D’s increased flexibility possibly contributed to the sampling of a wider range of structural states observed in RMSD and Rg. F177D was characterized by the most expansive and diffuse energy basin, reflecting a wider conformational space. A larger proportion of these conformations, however, occupied higher-energy regions, suggesting reduced structural stability and possibly supporting the observed loss of activity despite preservation of the overall energy landscape. Despite exhibiting a relatively restricted conformational space and predominantly occupying low-energy conformations, Y66E displayed reduced activity and thermostability. These results suggest that the global conformational stability captured by RMSD, Rg, and FEL analyses may not fully reflect local structural perturbations that influence enzyme function. Similarly, the decreased activity observed in the other variants despite stable RMSD and Rg profiles indicates that preservation of the overall protein fold alone is insufficient to maintain catalytic competence. Instead, local structural features and dynamic properties are likely to play a critical role in enzyme function. It is possible that the mutations had an allosteric effect, perturbing local hydrogen-bonding networks or altering the centrality of key residues involved in intramolecular communication, thereby affecting catalysis despite maintaining overall structural stability.

Although improvements in solubility were achieved through both fusion tags and surface hydrophobicity reduction, the accompanying effects on activity and thermostability highlight the complexity of protein engineering. Nevertheless, these findings demonstrate the importance of protein surface properties in governing aggregation behavior and provide valuable insights for the future design of soluble and functional carbonic anhydrases. For industrial applications, improved soluble production may ultimately provide greater practical benefit than maximizing specific activity alone, as increased enzyme yield can compensate for moderate reductions in catalytic efficiency. Future work should therefore employ high-throughput screening of engineered variants to identify mutation and fusion-tag combinations that provide an optimal balance between soluble expression, catalytic activity, and thermostability.

## 4. Materials and Methods

### 4.1. Sequence Analysis and Surface Patch Characterization

The amino acid sequence of the CA from *H. thermophila* was retrieved from the NCBI database [50] (NCBI Accession number: WP_317066981.1). A three-dimensional dimeric structure of the protein without the signal peptide was generated using homology modeling in SWISS-MODEL (https://swissmodel.expasy.org/, Accessed on 13 October 2025) [51,52], employing the crystal structure of *Persephonella marina* CA (PDB ID: 6EKI) as a template [53]. The quality of the generated model was assessed using PROCHECK (https://saves.mbi.ucla.edu/ Accessed on 13 October 2025) [54] for stereochemical evaluation and Verify3D (https://saves.mbi.ucla.edu/, Accessed on 13 October 2025) [55] to confirm the compatibility of the atomic model with its amino acid sequence.

The validated structure was subsequently imported into the MOE v2024.06 software (Chemical Computing Group ULC, Montreal, QC, Canada) for further structural analysis. Prior to analysis, the model was prepared by adding hydrogen atoms, assigning appropriate protonation states, and performing energy minimization to relieve steric clashes and optimize geometry. Surface and electrostatic properties were then characterized using MOE’s built-in tools. Hydrophobic surface patches were identified based on residue hydrophobicity scales and solvent-accessible surface area calculations, allowing visualization of regions prone to aggregation.

Aggrescan3D v2.0 [15,56] was also used to probe the HtCA dimer for exposed aggregation-prone surface regions. The software integrates experimentally derived residue aggregation propensities with structural information to identify solvent-accessible hydrophobic patches that may promote aggregation. Residues associated with high aggregation propensity scores were considered potential targets for surface engineering aimed at improving protein solubility. The results from MOE and Aggrescan3D were integrated to identify candidate residues for mutagenesis, taking into account both their involvement in hydrophobic surface patches and their potential functional roles. A total of 5 mutations were chosen and produced in vitro.

### 4.2. Bacterial Strains and Construction Vector Expression

The gene encoding HtCA, along with sequences encoding the B1 domain of protein G (Gb1), the T7B9 tag peptide, and the engineered solubility-enhancing tag ng3-NEXT, were obtained from Integrated DNA Technologies (IDT, Leuven, Belgium). All genes were codon-optimized for production in *E.coli*. Primers containing Gibson Assembly-compatible overhangs were designed for all constructs and synthesized by the same provider. The HtCA gene and each fusion tag sequence were amplified separately by PCR using a high-fidelity DNA polymerase. The amplified fusion tag fragments (Gb1, T7B9, and ng3-NEXT) were subsequently assembled with the HtCA gene, positioned at the C-terminus of HtCA and cloned into the pETM10 expression vector using the Gibson Assembly method [57]. The resulting constructs were named HtCA-T7B9, HtCA-Gb1, and HtCA-NEXT.

Site-directed mutagenesis was performed to introduce specific point mutations into HtCA using PCR-based overlap extension. All constructs had a His-tag on the C-terminal of the protein sequence. All protein sequences and oligonucleotide primers used in this study are listed in Supplementary Tables S1 and S2. *E. coli* NM522 was used for cloning procedures, while *E. coli* BL21(DE3) was employed for recombinant protein expression.

### 4.3. Protein Expression, Protein Purification and SDS-PAGE

Colonies of *E. coli* BL21(DE3) containing the recombinant genes for HtCA and its variants were inoculated into LB medium with 50 µg/mL kanamycin. Starter cultures were grown overnight at 37 °C with shaking at 250 RPM and thereafter used to inoculate 500 mL LB medium with 50 µg/mL kanamycin. Cultures were grown at 37 °C with 250 RPM shaking until the OD600 reached 0.6. Thereafter, IPTG and ZnSO4, both at a final concentration of 0.1 mM, were added to the cultures and further cultured for another 4 h. Cells were harvested by centrifugation (5000× *g* for 15 min) and the pellet was then resuspended in lysis buffer for an hour prior to sonication. Sonication was performed at 70% amplification for 6 min (5 s on, 5 s off per cycle) on ice, and the lysate was spun down at 4 °C for 10 min at 3000× *g* to remove cell debris. Ni2+ affinity chromatography was used to purify the enzyme in the supernatant using the Ni-NTA resin affinity column (QIAGEN GmbH, Hilden, Germany). All proteins were subjected to SDS-PAGE to determine their molecular mass and purity. Samples were mixed with 5× Laemmli loading buffer and β-mercaptoethanol and heated at 95 °C for 5 min before being loaded onto a 12-well SDS-PAGE pre-cast 12% Mini-PROTEAN^®^ TGX Stain-Free™ gel (Bio-Rad Laboratories, Hercules, CA, USA) alongside a Precision Plus Protein™ Unstained Bio-Rad protein marker (Bio-Rad Laboratories, Hercules, CA, USA) with a range of 20–250 kDa. Gels were activated in the Bio-Rad Gel Doc XR Imaging System (Bio-Rad Laboratories, Hercules, CA, USA) for 5 min.

### 4.4. CO_2_ Hydration Assay

CO_2_ hydration activity was measured using the Wilbur–Anderson assay [58], and all assays were performed on ice. A CO_2_-saturated solution was prepared using dry ice and used as the substrate. To a Tris-Cl buffer (25 mM) of 3 mL, 50 µL of enzyme solution was added and the reaction started by the addition of 2 mL of the CO_2_ saturated solution. The time for a pH drop from 8.4 to 6.4 was recorded for all reactions, including the blank, and the WAU was calculated as follows:

(T0 − T1)/T1(1)

where T0 is the time (s) it takes for the blank reaction to reach pH 6.4 and T1 is the time for the enzyme-driven reaction. As protein concentrations were not normalized prior to activity assays, specific activity was calculated by normalizing WAU to the protein concentration of each sample and expressed as WAU/mg.

### 4.5. Thermostability Assays

The thermostability of HtCA and its variants was evaluated by incubating protein samples at temperatures ranging from 0 °C to 90 °C. Aliquots were incubated for 1 h at the specified temperatures using a thermocycler (Bio-Rad Laboratories, Hercules, CA, USA) to ensure precise temperature control. Following incubation, samples were immediately cooled on ice to halt further thermal denaturation. Residual enzyme activity was determined using the CO_2_ hydration assay and compared to that of an unheated control. Thermostability was expressed as the percentage of residual activity relative to the control, which was defined as 100% activity.

### 4.6. MD Simulations and Structural Analysis

MD simulations were performed using GROMACS (version 2024) [59]. The protein structures were prepared by adding hydrogen atoms and assigning appropriate protonation states in H++ [60]. The AMBER ff14SB forcefield [61] was employed for protein parameterization and the system was solvated in a cubic box using the TIP3P water model and neutralized by the addition of counterions. Parameters for the catalytic Zn^2+^ ion and coordinating residues were inferred using AmberTools22 (released 2022) [62,63]. Energy minimization was first carried out to remove steric clashes and optimize the system geometry. This was followed by equilibration in two phases: constant volume (NVT) equilibration and constant pressure (NPT) equilibration, each performed under periodic boundary conditions to stabilize temperature and pressure, respectively.

A single MD simulation was subsequently conducted for each system for 100 ns at 300 K. A simulation time of 100 ns was selected to provide adequate conformational sampling while maintaining computational efficiency. Simulation convergence was monitored using root mean square deviation of the protein backbone over the course of the trajectory. Trajectory analyses were performed using built-in GROMACS tools. Structural stability and flexibility were assessed by calculating the RMSD and root mean square fluctuation (RMSF), respectively. Principal component analysis (PCA) was performed using the GROMACS covariance analysis tool on the covariance matrix of backbone atomic fluctuations to identify the dominant conformational motions sampled during the simulation. The first two principal components obtained from PCA were used as reaction coordinates for Gibbs free energy landscape construction using the GROMACS gmx sham module.

## Figures and Tables

**Table 1:** List of residues in the patch clusters and the corresponding surface area.

Patch Cluster Number	Residues	Area (A)
1	T82, V84, **K91**, A93 P128, Y151, K221, I224, K228	290
2	A65, Y66 *, **V68, L70, V246, L248**	250
3	I74 *, F75 *, S76, Y77, F99, I100, V101, K106, M183, K190, I145, Y186, M213	310
4	H83, A87, L157, K160, K164, A165, V168, L171, L175, V176, F177 *, K180, K184, L230	340
5	E47, **M48**, I51, K110, **V134**, V136 *, I142, **V144, L199,** **T205**,	250
6	K33, E37, Y38, E41, L42	140

* Residues chosen for mutation. In bold are HtCA’s interface and catalytic pocket residues [31].

## Data Availability

The original contributions presented in this study are included in the article/Supplementary Material. Further inquiries can be directed to the corresponding author.

## Supplementary Materials

The following supporting information can be downloaded at: https://www.mdpi.com/article/10.3390/ijms27167498/s1.
