# Supplementary material for: Engineering of a Carbonic Anhydrase from Hydrogenimonas thermophila Through Fusion Tags and Surface Mutagenesis Enhances Solubility While Revealing Stability–Function Relationships
Source: Int J Mol Sci. 2026 Aug 21;27(16):7498. doi: 10.3390/ijms27167498 (PMC13513295; doi:10.3390/ijms27167498)
Supplement: Supplementary file 1 [file ijms-27-07498-s001.zip › ijms-4460755-supplementary.pdf]

Supplementary Data:

Engineering of a Carbonic Anhydrase from *Hydrogenimonas thermophila* Through Fusion Tags and Surface Mutagenesis Enhances Solubility While Revealing Stability–Function Relationships

Colleen Varaidzo Manyumwa [1], Carsten Jers [1] and Ivan Mijakovic [1,2,*]

[1] Novo Nordisk Foundation Biotechnology Research Institute for the Green Transition, Technical University of Denmark, 2800 Kongens Lyngby, Denmark; covama@dtu.dk (C.V.M.); cjer@dtu.dk (C.J.)
[2] Department of Life Sciences, Chalmers University of Technology, SE-41296 Gothenburg, Sweden
[*] Correspondence: ivan.mijakovic@chalmers.se

Supplementary Table S1. Sequences of solubility tags introduced to HtCA.

Tag	Protein Sequence
HtCA (signal peptide underlined)	<u>MKKLLSLVAGMVLSTTIANAGVHEDV</u>HWGYSGKSAPEYWGELSPKYEMCKIGKNQSPVDIRTQDAYDVNLEQLIFSYYAKTTHVSKAHGIKVAVDPGNFIVVDGNKFKLKQFHFHSPSETMVNGRSFPLEAHFVHVSDDGQIAVIAVFFEYGKSNPLLEKIFSKAPAVENLDSSLVFKPKEIMKLYPANKEYYRYNGSLTTPPCTEGVRWIVMKKPLTVSKKQIKRFKELVPYSNNRPVQPINARVILK
ng3-NEXT	HGNAVQHSNAPLIDLGAEMKKQHKEAAPEGAAPAQGKAPAAEAKKEEAPKPKPVVGGGGSGGGGS
Gb1	TYKLILNGKTLKGETTTEAVDAATAEKVFKQYANDNGVDGEWTYDDATKTFTVTE
T7B9	LEDPEEASVTSTEETLTPAQEAAETEAANKARKEAELEAETAEQ

Supplementary Table S2. Sequence of primers used for recombinant CA construction and mutagenesis.

Gene/Mutation	Forward Primer (5′-3′)	Reverse Primer (3′-5′)
HtCA	TAAGAAGGAGATATACCATGGAGGACGTGCATTGGGGCTA	CAGTGGTGGTGGTGGTGGTGCTTCAAGATCACACGTGCATTG
Y66E	AAGACGCCGAAGATGTGGATTTAGA	AATCCACATCTTCGGCGTCTTGA
I74E	AGAACAGTTAGAATTCTCGTATTACGCTAAAAC	GCGTAATACGAGAATTCTAACTGTTCTAAATC
F75D	GAACAGTTAATCGATTCGTATTACGCTAAA	AGCGTAATACGAATCGATTAACTGTTCTAA

V136D	TTCGTTCATGATTCGGATGATGG	CATCATCCGAATCATGAACGAAAT
F177D	CAGTAGTTTAGTGGATAAGCCTAAA GAGATAATG	TCTCTTTAGGCTTATCCACTAAACTAC TGTCCAGA

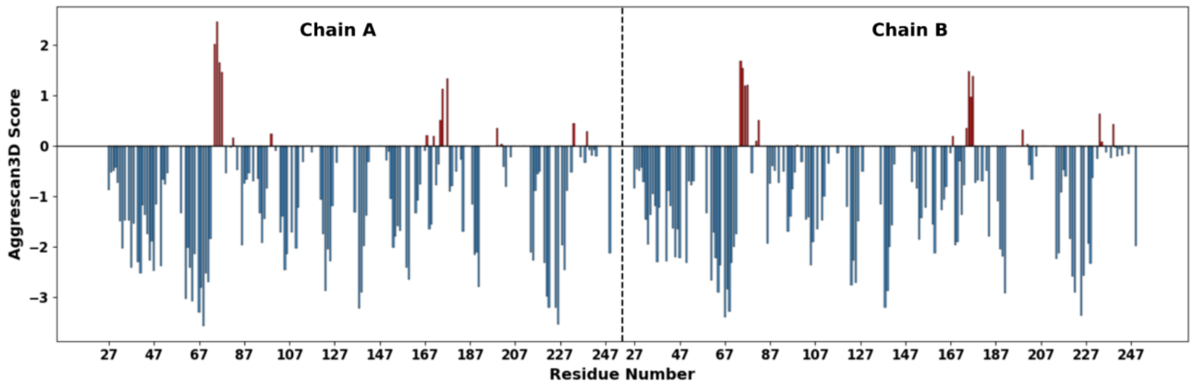

Supplementary Figure S1. Aggrescan3D analysis of the HtCA dimer showing residue-specific aggregation propensity scores. Positive scores (red) denote aggregation-prone residues, while negative scores (blue) indicate lower aggregation propensity. Residues I74 and F75 displayed the highest aggregation scores in both chains.
